# Editorial

**Published:** 1870-08

**Authors:** 


### Editorial.

Chicago Medical Society.—At the regular meeting on July 11th, Dr. Holmes presented a specimen of encephaloid disease of the orbit, removed from a boy 12 years of age. The case was one of special interest on account of the rarity of its occurrence in one so young. A gradual enlargement and some pain in the orbit was first noticed, after a blow received while playing base ball. In six weeks from the date of the injury, the swelling had increased until a mass the size of a teacup protruded from the orbit, surrounding and involving the eyeball. Upon removal, the mass presented a transparent jelly-like structure.

Dr. Holmes also presented a specimen of calcarious deposit in the choroid, removed from a patient 40 years of age. The eye had been destroyed by glass when the patient was six years old, and lately it had caused him much severe pain. The Doctor had in his possession a similar specimen, removed several years ago, which had caused the patient severe neuralgic pains for three years.

Dr. Davis presented a specimen of schirrhus, disease of the liver. The organ, at the time of its removal, weighed seven pounds. The cancerous growth involved full two-thirds of the right lobe, while a smaller mass was present in the left lobe. Both masses presented a retraction in the center, similar to that of the nipple in cancer of the breast. A specimen of the cancerous tissue was exhibited under the microscoɪ e, presenting the appearance of a simple aggregation of small granular cells, and an occasional fibrous shred. A number of specimens from different parts of the cancerous masses had been carefully examined under the microscope, but without the detection of anything more than was shown in the specimen exhibited. None of the oval nucleated cells described as characteristic of schirrhus growths could be found in any of the specimens. The case at first presented all the symptoms of chronic gastritis, without any enlargement of the liver. After the hardening and enlargement of the organ had once begun, the growth was rapid, and the patient soon died. The account of another somewhat similar case was also given by the Doctor.

Dr. Fitch mentioned a case that had came under his notice in which the disease was supposed to be hereditary. The patient here also complained most of intense burning pain in the stomach.

Dr. Fitch also presented a case of imperforate rectum in a child otherwise well formed at birth. An operation was performed, but the child died within a few days. Dr. F. suggested whether, in such cases, it was not preferable that the child should die, rather than that it should live with a disgusting artificial anus.

---

Professorial Changes.—Prof. E. Gilbert Wheeler, who had filled the two chairs of Chemistry in the Chicago Medical College, having resigned, the chair of Inorganic and Analytical Chemistry has been filled by the appointment of F. Gray Bartlett, Ph.D., of this city; and that of Organic Chemistry and Toxicology, by the appointment of H. P. Merriman, M.D., also of this city. A new chair of Ophthalmology and Otology has been created and filled by the appointment of Sam'l J. Jones, M.D.

These are excellent appointments, each appointee having special qualifications for the performance of the duties assigned to him.

---

LITY FOR THE MONTH OF JUNE, 1870.

| | | | | | |
|---|---|---|---|---|---|
| Accident, by aconite | 1 | Diarrhœa, chronic | 3 | Lungs, congestion | 4 |
| " drowned | 6 | Diphtheria | 7 | " hemorrhage | 1 |
| " fracture of skull | 2 | Dropsy, general | 4 | Manslaughter | 2 |
| " by fall | 2 | " of chest | 1 | Malformation, general | 2 |
| " gasoline | 2 | Dysentery | 16 | Mouth-canker sore | 1 |
| " lightning | 4 | " acute | 1 | Measles | 10 |
| " railroad | 4 | Empyemia | 1 | " & complications | 7 |
| Abscess | 1 | Entero-colitis | 1 | Metro-peritonitis | 2 |
| Albuminuria | 1 | Enteritis | 9 | Meningitis | 14 |
| Apoplexy | 6 | Epilepsy | 1 | " cerebro-spinal | 2 |
| Apthæ | 1 | Erysipelas | 1 | " tubercular | 2 |
| Asphyxia | 1 | Fever, congestive | 1 | Old age | 6 |
| Births, premature | 18 | " puerperal, | 4 | Paralysis | 7 |
| " still | 48 | " remittent | 2 | Pericarditis | 1 |
| Bowels, constipation | 1 | " scarlet | 28 | Peritonitis | 2 |
| " congestion | 1 | " " complications | 9 | " puerperal | 1 |
| " hemorrhage | 1 | " " malignant | 2 | Pneumonia | 10 |
| " ulceration | 1 | " typhoid | 10 | " broncho | 1 |
| Brain, congestion | 9 | Gastritis | 1 | " & complications | 2 |
| " inflammation | 11 | Gastro-Enteritis | 2 | Pyæmia | 1 |
| " softening | 1 | Hæmatemesis | 1 | Rheumatism | 1 |
| Bronchitis | 5 | Hemorrhage of ulcers | | Scrofula | 2 |
| " chronic | 1 | on leg | 1 | Small-Pox | 5 |
| " capillary | 1 | Heart disease | 2 | Sunstroke | 8 |
| Cancer of Breast | 1 | " " and dropsy | 1 | Suicide, by drowning | 1 |
| " inguinal glands | 1 | " hypertrophy | 1 | " " strychnine | 1 |
| " " stomach | 2 | " fatty degenerat'n | 1 | " " hanging | 1 |
| " " uterus | 5 | " valvular disease | 4 | " " shooting | 1 |
| Childbirth | 1 | Hip-joint disease and | | Suffocation from tumor | 1 |
| Chorea | 1 | abscess | 1 | Tabes mesenterica | 20 |
| Cholera Infantum | 145 | Hepatitis | 1 | Teething | 10 |
| " morbus | 3 | Hernia, strangulated | 3 | " & complications | 9 |
| Consumption | 45 | Hydrocephalus | 6 | Tetanus | 1 |
| Convulsions | 90 | " acute | 11 | Thorax, malformation | 1 |
| Colitis | 1 | Inanition | 11 | Trismus | 1 |
| Croup | 12 | Icterus menonatorum | 1 | Tumor, ovarian | 1 |
| " membranous | 4 | Jaundice | 1 | Unknown | 2 |
| Cynanch tonsillaris | 1 | Kidneys, Bright's dis- | | Whooping-cough | 2 |
| Debility, general | 4 | ease | 1 | " & complications | 2 |
| Delirium Tremens | 1 | Laryngitis | 2 | | |
| Diarrhœa | 37 | Liver, degeneration | 1 | Total | 720 |

Deaths in June, 1870, 720 | Deaths in June, 1869, 434 | Increase, 286
Deaths in May, 1870, 436 | Increase, 284

AGES.

| | | | | | |
|---|---|---|---|---|---|
| Under 1 | 360 | 10 to 20 | 16 | 70 to 80 | 12 |
| 1 to 2 | 94 | 20 to 30 | 45 | 80 to 90 | 4 |
| 2 to 3 | 22 | 30 to 40 | 44 | 90 to 100 | 1 |
| 3 to 4 | 19 | 40 to 50 | 32 | Unknown | 5 |
| 4 to 5 | 12 | 50 to 60 | 18 | | |
| 5 to 10 | 23 | 60 to 70 | 13 | Total, | 720 |

Males, 387 | Females, 333 | Total, 720

Single, 599 | Married 121 | Total, 720

White, 714 | Colored, 6 | Total, 720

| | | | | | |
|---|---|---|---|---|---|
| Atlantic Ocean | 1 | England | 9 | Russia | 1 |
| Bohemia | 6 | France | 2 | Sweden | 6 |
| Canada | 4 | Germany | 73 | Scotland, | 5 |
| Chicago, Native | 114 | Holland | 3 | Switzerland | 2 |
| Chicago, Foreign | 346 | Ireland | 38 | Unknown | 6 |
| U. S., other parts | 86 | Norway | 12 | | |
| Denmark | 4 | Poland | 2 | Total, | 720 |

MORTALITY BY WARDS FOR THE MONTH.

| Wards. | Mortality. | Wards. | Mortality |
|---|---|---|---|
| 1 | 6 | 19 | 19 |
| 2 | 18 | 20 | 23 |
| 3 | 27 | Accidents | 21 |
| 4 | 13 | County Hospital | 12 |
| 5 | 19 | Home for Friendless | 5 |
| 6 | 40 | Hospital Alexian Brothers | 2 |
| 7 | 30 | Immigrants | 30 |
| 8 | 63 | Jewish Hospital | 3 |
| 9 | 62 | Lake Hospital | 1 |
| 10 | 17 | Manslaughter | 2 |
| 11 | 27 | Mercy Hospital | 7 |
| 12 | 18 | Soldiers' Home | 2 |
| 13 | 12 | St. Joseph Orphan Asylum | 8 |
| 14 | 13 | Suicide | 4 |
| 15 | 77 | | |
| 16 | 40 | Total, | 720 |
| 17 | 44 | | |
| 18 | 55 | | |

OBITUARY.—JOSEPH S. HILDRETH, M.D.—Dr. Hildreth died at his residence, No. 526 Wabash avenue, at 5 o'clock July 22.

The death of this eminent physician, who has been cut off thus suddenly in the very flower of his manhood, and in the midst of a career of usefulness and promise, will cause deep sorrow throughout a wide circle of personal friends, while it is to be equally deplored as a loss to the medical fraternity, of which the deceased was one of the brightest ornaments. Dr. Hildreth had been suffering from neuralgia for several days, and had resorted to various remedies for the alleviation of his suffering. On Thursday afternoon he suffered so intensely that he was induced to take chloroform. He inhaled it for a considerable time, and, about 9 o'clock in the evening, he took a dose of hydrate of chloral. While under the partially deranging influences of these combined remedies, he resorted to a still more powerful one—gelserum—in the hope that it would relieve him from his pain. Unhappily, he committed an error in judgment as to the quantity, and took an overdose. Dr. Bevan, Dr. Miller, and Dr. Byford were immediately called to his aid, but the poisonous influences were so strong that it was found impossible to arrest the effects of the drugs. The Doctor became insensible, and expired about 5 o'clock in the morning.

